# ELDER MISTREATMENT FOLLOW-UP: CONNECTING EMERGENCY DEPARTMENTS AND COMMUNITIES

**DOI:** 10.1093/geroni/igz038.291

**Published:** 2019-11-08

**Authors:** Alice Bonner, Kristin Lees-Haggerty, Debi Lang, Bree Cunningham, Jason Burnett, Kathy Greenlee

**Affiliations:** 1 Institute for Health Care Improvement, Boston, Massachusetts, United States; 2 Education Development Center, Waltham, Massachusetts, United States; 3 UMASS Medical School, Shrewsbury, Massachusetts, United States; 4 MA Executive Office of Elder Affairs, Boston, Massachusetts, United States; 5 UT Health, Houston, Texas, United States; 6 Greenlee Global, Lenexa, Kansas, United States

## Abstract

To effectively address elder mistreatment (EM) in the emergency department (ED) hospitals must have mechanisms that promote and, to the extent possible, ensure patient safety post-discharge. However, the realities of working within busy hospitals--limited staff time, financial resources, and EM-specific expertise--prevent many EDs from being able to dedicate staff for patient follow up or develop EM multi-disciplinary teams. The fourth core element of the NCAEM’s ED Care Model aims to address this need with a roadmap for leveraging existing community resources. The roadmap provides streamlined tools to help hospitals assess their needs, identify existing teams and resources in their community, and connect with Adult Protective Services and other organizations. In this presentation we will present these tools and share case examples from beginning stages of feasibility testing in hospitals across the US. We will discuss specific strategies for implementing the model in hospitals of differing types, sizes, and resource levels.

